# Novel Murine Model of Atherosclerosis Progression Induced by a Modified Paigen Diet

**DOI:** 10.3390/biomedicines13112736

**Published:** 2025-11-08

**Authors:** María del Rosario Álvarez-Valadez, Alejandrina Rodríguez-Hernández, Felipa Andrade-Urzúa, Saraí Limón-Miranda, Adriana Ceballos-Gutiérrez, Jorge Agustín Velasco-Gutiérrez, Armando Gamboa-Domínguez, Adolfo Virgen-Ortiz, Enrique Sánchez-Pastor

**Affiliations:** 1 Centro Universitario de Investigaciones Biomédicas, Universidad de Colima, Colima 28045, Mexico; rosario.alvarez@unison.mx (M.d.R.Á.-V.);; 2 Facultad de Medicina, Universidad de Colima, Colima 28040, Mexico; 3 Departamento de Ingeniería Química y Bioquímica, Tecnológico Nacional de México-Instituto Tecnológico de Colima, Villa de Álvarez 28976, Mexico; 4 Departamento de Ciencias Químico Biológicas y Agropecuarias, Facultad Interdisciplinaria de Ciencias Biológicas y de Salud, Unidad Regional Sur, Universidad de Sonora, Navojoa 85880, Mexico; 5 Departamento de Patología, Instituto Nacional de Ciencias Médicas y Nutrición Salvador Zubirán, Mexico City 14080, Mexico

**Keywords:** atherosclerosis, dyslipidemia, blood pressure, paigen diet, histopathology

## Abstract

**Background/Objectives**: To better understand the mechanisms involved in atherosclerosis, different models have been developed, but these fail when studying the progression of this disease. The purpose of this study was to standardize a new model of atherosclerosis progression in rats using Paigen-type modified atherogenic diet. **Methods**: The design included a control group (*n* = 16) and 64 rats with atherogenic Paigen-type diet subdivided into four subgroups with different doses (Athero 1, Athero 2, Athero 3, and Athero 4). The atherogenic diet was supplemented orally in sequential stages: 1) Hypervitaminic (1.5 mL/kg/day for 12 days) and 2) Hyperlipidic (48 days ad libitum). Blood pressure, heart rate, aortic histopathology, inflammatory biomarkers, and biochemical lipid and liver profiles were measured in all groups on days 30 and 60. **Results**: All Athero 1 rats were sacrificed due to a poisoning for vitamin D2 excess. Athero 2 rats were sacrificed at day 30 showing severe atherosclerotic lesions (grades V–VIII). Athero 3 rats showed mild lesions (I–IV) at day 30 and severe lesions (V–VIII) at day 60. Athero 4 rats showed mild lesions (I–IV) at days 30 and 60. Diet-dependent changes in blood pressure and heart rate were observed. Furthermore, glycemia, dyslipidemia, and liver profile were associated with the degree of atherosclerotic lesion. **Conclusions**: “Athero 3” atherogenic diet generates a stable model to study the progress of atherosclerosis in rats.

## 1. Introduction

Cardiovascular diseases are a group of disorders of heart and blood vessels and are considered the leading cause of death worldwide. They include diseases such as cardiac ischemia, stroke, cerebrovascular disease, cardiac failure, among others. Cardiac ischemia may result from occlusive complications caused by atherosclerosis [1]. Atherosclerosis is a chronic inflammatory disease that affects the arterial wall and increases the risk of occlusion when the conditions of atherosclerotic plaque progression are persistent [2,3].

Experimental models have been developed for the study of atherosclerosis using different species of animals; however, murine models predominate due to several characteristics, including ease of treatment administration and lower feeding and maintenance costs. Over time, several atherogenic treatments have been used to induce atherosclerosis in murine models, including high-fat diets, chemical induction, mechanical trauma, the combination of two of these strategies, and recently, editing and/or gene deletion [4,5,6].

Most models of atherosclerosis using different forms of induction coincide in the location of plaque formation in the aorta artery; however, the degree of lesions observed varies with a predominance of mild lesions [7,8].

One of the most widely used models to study atherosclerosis is genetically modified mice (Apo E^−/−^, LDLR^−/−^), although they have important disadvantages such as high economic cost, availability, limited tissue, and surgical difficulties due to the small size of the animal. In this context, we were interested in developing a low-cost, easy-to-replicate rat model that would allow us to clearly study the stages of disease progression so that in the future scientists could study and better understand the mechanisms involved in the progression of atherosclerosis. Paigen diet includes hypervitaminic and hyperlipidemic phases and it has proven effective in reproducing key features of human disease [7,9,10]. The hypervitaminic phase induces hypercalcemia, tissue mineralization, and early vascular changes, sensitizing the organism to atherogenic processes [11,12]. The hyperlipidemic phase prolongs hyperlipidemia and promotes lipid accumulation in the arteries by elevating low-density lipoproteins and reducing basal metabolism [13]. This combination of phases provides a robust model of atherosclerosis, replicating both the biochemical and structural changes observed in human disease. Therefore, the purpose of this study was to standardize a new model of atherosclerosis progression in Wistar rats using Paigen-type modified atherogenic diet.

## 2. Materials and Methods

### 2.1. Animals

Male Wistar rats with an average weight of 200 ± 10 g were included in the present study. Animals were housed in pairs (two rats per polyether sulfone cage, with a floor area of 483.14 cm^2^ per animal) in a room with temperature of 22–25 °C with 45–60% relative humidity and 12-12 dark-light cycles. Food and water were provided ad libitum, with daily cage cleaning and bedding material changes to minimize environmental stress. Drinking water was always available for the animals to drink freely. All study protocols were approved by the Bioethics Committee of the University Center for Biomedical Research of the University of Colima (Project code 2021-7DC; approval date 9 September 2021) and the Internal Committee for the Care and Use of Laboratory Animals of the University Center for Biomedical Research of the University of Colima (Project code 2021-09; approval date 21 September 2021). All methods were conducted in accordance with the Guide for the Use of Laboratory Animals (July 1996) and NOM-062-ZOO-1999 on bioethics and care of experimental animals and reported according to ARRIVE guidelines [14].

### 2.2. Chemicals

Ergocalciferol (vitamin D2), cholesterol, thiouracil, and cholic acid were obtained from Sigma-Aldrich (Sigma-Aldrich, St. Louis, MO, USA). Olive oil (Carbonell special selection extra virgin, The Olive Oil Company, Deoleo^®^, Cordoba, Spain) and butter (with vegetable oil) were used. The experimental animals were fed a Standard diet (Rodent Laboratory Chow 5001, Purina^®^, Mexico City, Mexico). Kits for glucose, triglycerides, cholesterol (Total, HDL, and LDL), lactate dehydrogenase (LDH), alkaline phosphatase (ALP), alanine aminotransferase (ALT), and aspartate aminotransferase (AST) were obtained from SPINREACT^®^ (SPINREACT, Girona, Spain).

### 2.3. Experimental Design

**Figure 1:**
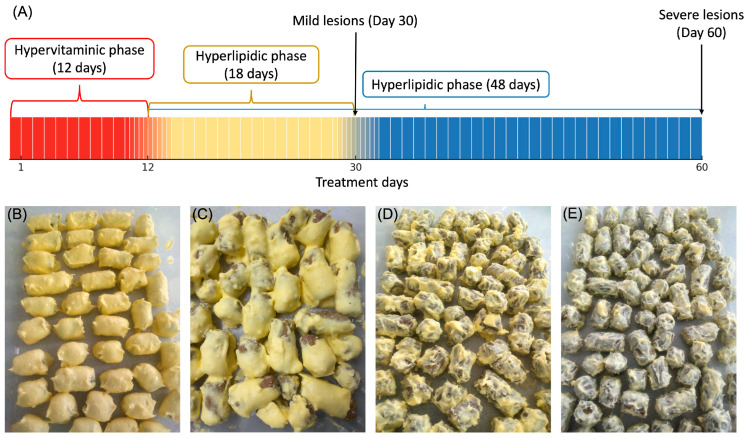
Experimental design showing the duration of hypervitaminic and hyperlipidic phases (**A**) and illustrative images of atherogenic diets in hyperlipidic phase, (**B**) Athero 1, (**C**) Athero 2, (**D**) Athero 3, and (**E**) Athero 4. The percentage of the components added to the standard diet was highest for Athero 1 and progressively decreased to Athero 4, as shown in Table 1.

Eighty rats were divided into five experimental groups as follows: one healthy control (*n* = 16) with standard diet and four (groups Athero 1, Athero 2, Athero 3, and Athero 4) treated with atherogenic “Paigen type” diet with modifications (*n* = 16 per group). This diet consisted of two sequential phases as follows: 1) Hypervitaminic, administered orally at a dose of 1.5 mL/kg/day, and 2) Hyperlipidic, administered *ad libitum* (Table 1). These five groups were subdivided on day 30 and day 60 of the treatment. The experimental design and details of the doses used for each group are shown in Table 1 and Figure 1A. The hypervitaminic suspension for the first phase of the diet was prepared daily and administered at approximately the same hour each day. Regarding the hyperlipidic diet, in this phase the components of the diet were mixed uniformly and then added to the outside of the standard feed cubes for rodents (Figure 1B–D).

### 2.4. Extraction of Aorta Segments

Rats were anesthetized with pentobarbital sodium (45 mg/kg body weight, i.p.) and sacrificed by exsanguination. The descending thoracic aorta was dissected and placed in Krebs–Henseleit solution (118 mM NaCl, 5 mM KCl, 1.2 mM MgSO_4_, 1.2 mM KH_2_PO_4_, 25 mM NaHCO_3_, 2 mM CaCl_2_, and 2 g of glucose; pH = 7.4) bubbled with a carbogen gas mixture (95% O_2_ and 5% CO_2_). The aorta segments from all rats were isolated and cleaned from adherent adipose and connective tissues and fixed in 10% neutral buffered formalin.

### 2.5. Histological Analysis and Atherosclerotic Lesions Classification

The aorta was divided into aortic rings of 2–3 mm, paraffin embedded, and 3 rings of 3 μm were prepared on silanized slides. Samples were dewaxed, rehydrated, and hematoxylin-eosin stained for classification according to the complement of two systems, a qualitative (morphologically classifies the progression of atherosclerotic lesions, based on the modified Stary classification, expanded from six to eight types of atherosclerotic lesions) [15] and a semi-quantitative system (morphologically classifies atherosclerotic lesions with the aim of differentiating between stable plaques and unstable/vulnerable plaques, with lesions divided into four types) [16]. Based on the histological characteristics present in the arterial wall, arteries were classified as normal, with mild or severe atherosclerotic lesions. They were classified as normal arteries when they exhibited an arterial wall thickness of approximately 90 µm, with continuous lamellae, without extracellular lipid deposits, focal endothelial lesions, fibrous caps, calcium clusters, lipid cores, hematomas–hemorrhages, or thrombi. Atherosclerotic lesions were classified as mild when they exhibited increased arterial wall thickness, fatty streaks, small extracellular lipid deposits, extracellular lipid cores, and some focal endothelial erosions. Finally, they were classified as severe atherosclerotic lesions when the aortic ring wall showed increased thickness, lipid accumulation of vacuolated macrophages, fibroatheromas, calcium deposition in the atherosclerotic plaque, focal endothelial erosions, vascular hypertrophy and remodeling, hematomas–hemorrhages, or thrombi. Note: Due to decalcification, empty spaces do not interfere with the classification, because calcium deposits can easily be recognized in H-and-E staining showing dark purple deposits.

Some aortic sections were immunolabeled for alpha-smooth muscle actin (α-SMA) CD45 or CD68 as inflammatory markers. For immunolabeling, Actin, Smooth Muscle (1A4) Mouse Monoclonal Antibody (1:500), CD68 (Kp-1) Mouse Monoclonal Antibody (1:500) (Cell Marque, Rocklin, CA, USA), or Mouse Monoclonal Anti-CD45 antibody (1:2000) (Bio SB, Santa Bárbara, CA, USA) were used. Detection was performed using the ImmunoDetector DAB HRP Brown Immunohistochemistry system (Bio SB, Santa Bárbara, CA, USA) was used. Images were captured with an Axiocam MRC-5 (Zeiss, Oberkochen, Germany) attached to an AxioPlan 2 microscope (Zeiss, Oberkochen, Germany). Negative control was included by omitting primary antibodies.

### 2.6. Biochemical Analysis

Blood samples were collected intracardially in tubes without clot activator or gel BD Vacutainer^®^ (Becton, Dickinson and Company, East Rutherford, NJ, USA), for the measurement of total cholesterol (TC), triglycerides, high-density lipoprotein (HDL), low-density lipoprotein (LDL), glucose, aspartate aminotransferase (AST), alanine aminotransferase (ALT), lactate dehydrogenase (LDH), and alkaline phosphatase (ALP). Biochemical parameters such as glucose, serum lipid profile, and serum levels of liver biochemistry panels were measured using the enzymatic colorimetric test kit Spinreact^®^ (SPINREACT, Girona, Spain; TC ref. kit: 41022; triglycerides ref. kit: 41032; HDL ref. kit: 1001097; LDL ref. kit: 41023; Glucose ref. kit: 41012; AST ref. kit:1001160; ALT ref. kit: 1001170; LDH ref. kit: 1001260; ALP ref. kit: 1001130) according to the manufacturer’s instructions.

### 2.7. Blood Pressure

Systolic pressure (SP), diastolic pressure (DP), and heart rate (HR) were measured in weeks one, four, and eight. The rats were placed into a cylindrical restrainer the week prior to the experiments to reduce stress and acclimate into the system (30 ± 1.5 °C). The blood pressure of rats was measured by tail-cuff method using a non-invasive Mouse and Rat Blood Pressure System (IITC Life Science Inc., Woodland Hills, CA, USA). Five recordings were made with a 1 min rest between each recording, and the average blood pressure was calculated for each rat.

### 2.8. Statistical Analysis

Statistical analysis was performed using the Graph Pad Prism Software version 7.02. The normality of the distribution was confirmed using the Shapiro–Wilk test. Based on the results obtained from the normality test, one way analysis of variance (ANOVA) test and Tukey post hoc test were used for comparison between groups. For all analysis, *p* < 0.05 was considered statistically significant. Quantitative data were expressed as the mean ± SEM.

## 3. Results

### 3.1. Clinical Manifestations in Rats During Induction Phase

The induction phase of the atherosclerotic model was for a period of two months; clinical manifestations were analyzed in all study groups, and the results are shown in Table 2. The Atherogenic diet caused severe complications in groups Athero 1 and Athero 2. Therefore, it was decided to euthanize the rats (in accordance with the humanitarian endpoints of the NOM-062-ZOO-1999) to prevent unnecessary suffering.

### 3.2. Blood Pressure Responses to Atherogenic Diet

**Figure 2:**
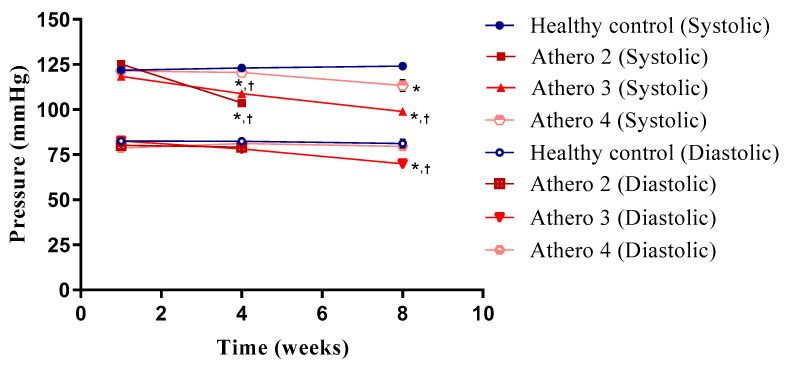
Comparison of the effect of atherogenic diets versus normal diet on systolic and diastolic blood pressure in Wistar rats for a period of two months. * Statistically significant regarding the healthy control (*p* < 0.05). ^†^ Statistically significant regarding the mild lesions (*p* < 0.05). The values are expressed as the means ± SEM (*n* = 8 for each group). Intergroup analysis: *t* test.

To determine the effect of each type of diet and its interaction with time (different phases of atherosclerosis) on blood pressure and heart rate, these parameters were monitored for each experimental group in weeks 1, 4, and 8 (Figure 2).

In the healthy control group, systolic and diastolic blood pressure, as well as heart rate, showed no significant changes during the 8 weeks of the protocol; the values obtained in this study using an indirect method for their measurement are in the normal range reported for Wistar rats. Groups Athero 2, Athero 3, and Athero 4 did not show significant differences in any of the parameters at the beginning of the treatment with respect to the control group. In week 4, groups Athero 2 and Athero 3 showed a significant decrease in SP and HR compared to the control group and the initial week of each group. Group Athero 4 did not show changes in SP and DP after weeks. At week 8, SP in group Athero 4 significantly decreased in comparison with the control group at the same time point, meanwhile group Athero 3 experienced a significant decrease in SP and DP compared to the control group.

**Figure 3:**
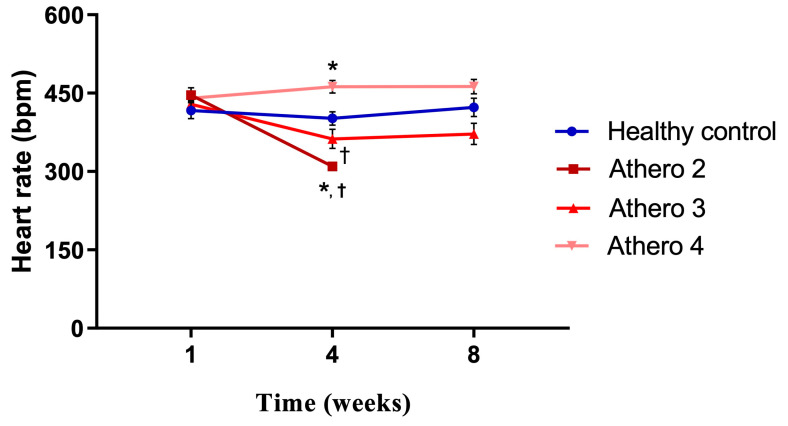
Effect of atherogenic diets on heart rate in Wistar rats. * Statistically significant regarding the healthy control (*p* < 0.05). ^†^ Statistically significant regarding the mild lesions (*p* < 0.05). The values are expressed as the means ± SEM (*n* = 8 for each group). Intergroup analysis: *t* test.

When analyzing the HR (Figure 3), no significant differences were observed between the experimental groups at the beginning and at the end of the study; however, in week 4 the HR increased in group Athero 4 and decreased in groups Athero 2 and 3 with respect to the control group. These changes were statistically significant.

### 3.3. Histopathological Analysis

**Figure 4:**
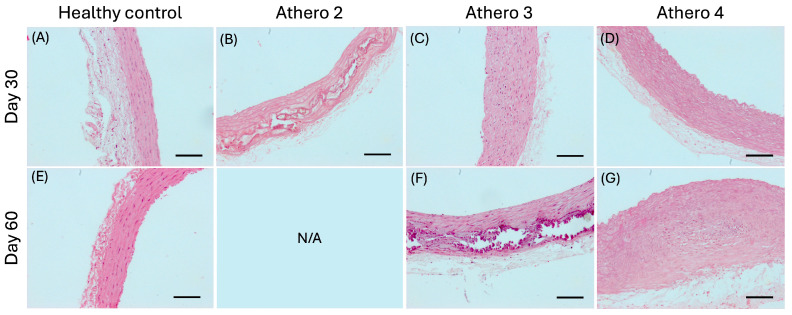
Illustrative images of the histopathological changes in the thoracic aorta in hematoxylin and eosin-stained sections from all experimental groups. (**A**,**E**) Healthy arteries from control rats at days 30 and 60. (**B**) Severe lesions in aortic ring from Athero 2 group at day 30. (**C**,**F**) Aortic rings from Athero 3 group progressed to mild lesions after 30 days and severe atherosclerotic lesions at day 60, respectively. (**D**,**G**) Arteries from Athero 4 group progressed to mild lesions after 30 and 60 days of treatment. Images magnified by 10×. N/A: Not applicable, due to loss of experimental animals. Scale bar 100 µm.

To determine the atherosclerotic stage of the rats in each experimental group, the descending aorta was removed, and the degree of atherosclerosis was determined by histological analysis. Figure 4A and Figure 4E show the characteristics of the aortic rings from the control group corresponding to a healthy arterial wall on day 30 and day 60 (normal arterial wall thickness (88.49 and 92.62 μm, respectively), with continuous lamellae, and without extracellular lipid deposits, focal endothelial lesions, fibrous caps, calcium deposits, lipid cores, hematomas–hemorrhages, or thrombi). In group Athero 2, the grade of lesions classified was severe on day 30 (the aortic ring wall exhibited a thickness of 145.55 µm, lipid accumulation of vacuolated macrophages, fibroatheromas, calcium deposition in the atherosclerotic plaque, focal endothelial erosions, and vascular hypertrophy and remodeling; see Figure 4B). Group Athero 3 exhibited characteristics of mild lesions on day 30 (showing an increase in arterial wall thickness to 175.03 µm, fatty streaks with small extracellular lipid deposits, extracellular lipid core, and some focal endothelial erosions; see Figure 4C) and progressed to severe atherosclerotic lesions by day 60 (with an aortic wall thickness of 163.19 µm, fibroatheromas, fibrous caps, calcium deposition in the atherosclerotic plaque, focal endothelial erosions, and vascular hypertrophy and remodeling; see Figure 4F). Finally, group Athero 4 had characteristics corresponding to mild lesions on day 30, and although these characteristics increased and intensified, the lesions were still classified as mild on day 60 (with an arterial wall thickness of 148.14 and 308.93 µm, respectively, fatty streaks with extracellular lipid deposits, extracellular lipid core, and some focal endothelial erosions; see Figure 4D and Figure 4G).

Unlike groups Athero 2 and Athero 4, group Athero 3 showed atherosclerotic plaques in the aortic rings of mild lesions on day 30 and of severe lesions on day 60, consistent with what is expected from an atherosclerosis induction model. Therefore, group Athero 3 was selected to perform the following determinations. Once the ideal atherogenic diet (group Athero 3) was characterized for the development of atherosclerosis progression, we worked with the following 2 groups: a healthy control (*n* = 8) and an atherosclerotic group (Athero 3; *n* = 16).

**Figure 5:**
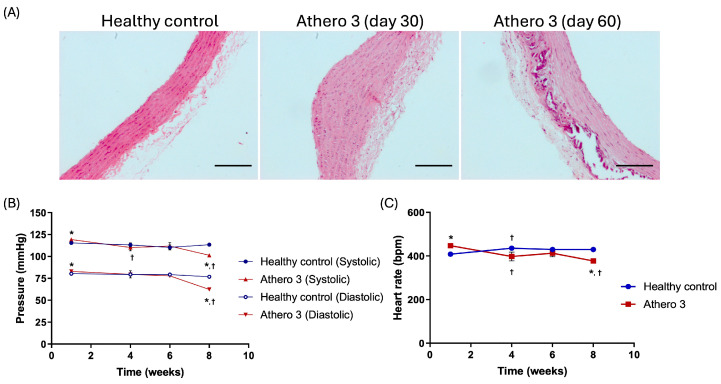
(**A**) Illustrative images of the histopathological changes in the thoracic aorta in hematoxylin and eosin-stained sections from group Athero 3. Images magnified by 10×. (**B**) Effect of the atherogenic diet versus normal diet on systolic and diastolic blood pressure in Wistar rats. (**C**) Effect of the atherogenic diet on heart rate in Wistar rats. * Statistically significant regarding the healthy control (*p* < 0.05). ^†^ Statistically significant regarding the mild lesions (*p* < 0.05). The values are expressed as means ± SEM (*n* = 8 for each group). Intergroup analysis: *t* test. Scale bar 100 µm.

Histopathological analysis in the groups was consistent with the standards established for the diet (Figure 5A), where the control group showed characteristics corresponding to a healthy arterial wall, with a thickness of 93.77 µm, continuous lamellae, no extracellular lipid deposits, focal endothelial lesions, fibrous caps, calcium deposits, lipid cores, hematomas–hemorrhages, or thrombi. In group Athero 3, mild lesion characteristics were observed on day 30, including an arterial wall thickness of 203.8 µm, fatty streaks with small extracellular lipid deposits, extracellular lipid core, and some focal endothelial erosions; by day 60, the lesions were classified as severe, with an aortic wall thickness of 155.27 µm, fibroatheromas, fibrous caps, calcium deposition in the atherosclerotic plaque, focal endothelial erosions, and vascular hypertrophy and remodeling. The arterial wall thickness was quantified from at least eight rats in each group; the average and the statistical analysis is shown in Table 3. These histopathological changes in the aorta (Figure 5A) and SP, DP, HR (Figure 5B,C) were monitored, confirming the reproducibility of the model.

**Figure 6:**
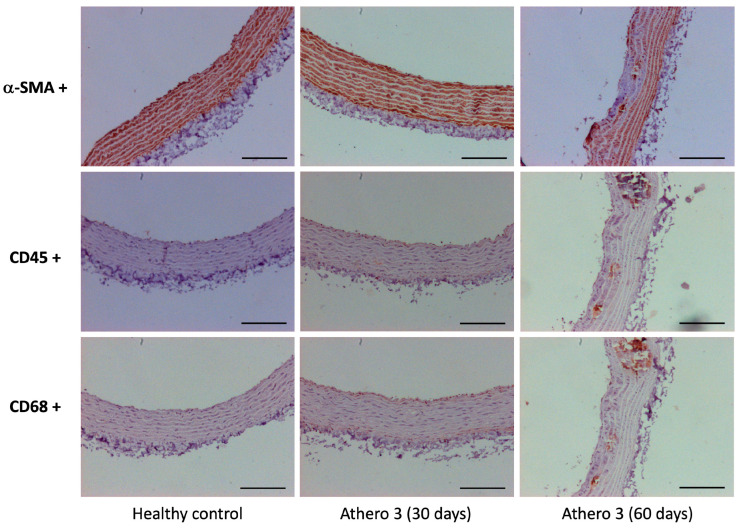
Immunohistochemical staining of aortic ring sections. Illustrative images from the healthy control group, the atherosclerosis group after 30 days of atherogenic diet, and the group after 60 days on the diet are presented. Samples from both healthy and atherosclerotic groups were immunolabeled for α-SMA, CD45, and CD68, with positive expression of each marker indicated in brown. All images were taken at 10× magnification. Three different experiments were performed for each condition. Scale bar 100 µm.

Further analysis of plaque progression to confirm the plaque composition was carried out by labeling α-smooth muscle actin (α-SMA) and the biomarkers of inflammation CD45 and CD68 (Figure 6). The images from the immunocytochemical study of the aortic wall indicate that at 30 days of atherosclerosis development, there is an increased presence of smooth muscle cells. As the disease progresses, deterioration is observed by day 60. CD45+ staining reveals positive expression on the endothelial surface with minimal expression in the adventitia of the aortic wall of rats after 30 days of an atherogenic diet, and by 60 days, positive expression is predominantly localized in the areas of atheroma formation. Additionally, CD68+ staining for macrophage localization shows that by day 30, there is already immunoreactivity present in the endothelial surface and in the adventitial layer of the aortic wall in the group that received an atherogenic diet. By day 60, immunoreactivity is more focused in the regions of atheroma formation.

### 3.4. Effects of the Atherogenic Diet on Lipid and Hepatic Profile, and Glucose Serum Levels

As part of the characterization of the atherosclerotic model, metabolic markers were determined for each group (healthy control, atherogenic group Athero 3 on day 30 (mild lesions), and atherogenic group on day 60 (severe lesions)). An increase in the serum lipid profile (triglycerides, total cholesterol, LDL, and HDL) was found in the atherosclerotic groups when compared to the control (Table 4). Also, the atherosclerotic groups showed a significant increase in glucose levels, which partly coincides with lipid profile levels. Serum liver markers showed an increase dependent on the time of treatment, which may be related to the atherosclerotic degree of the lesions present in each group. Overall, these findings support our model as a very suitable approach to study the different stages of atherosclerosis in rats, which might be more accessible for researchers.

## 4. Discussion

Developing new experimental animal models that mimic the progression of atherosclerosis and its clinical manifestations is important for the design of new studies aimed at better understanding all the mechanisms associated with this disease and proposing new therapeutic targets for its treatment. In the present study, we set out to standardize a new model of atherosclerosis in rats in which the phases of disease progression are clearly identified. For induction we used an atherogenic Paigen-type diet with modifications. The two-phase design of the atherogenic diet is based on progressively inducing the metabolic and structural conditions necessary for the development of atherosclerosis. In the first phase, the oral administration of vitamin D_2_ combined with cholesterol and olive oil aimed to generate an initial state of hypercalcemia, tissue mineralization, and dyslipidemia—conditions that predispose to the formation of early vascular lesions [11,12]. Subsequently, in the second phase, the ad libitum supply of high concentrations of cholesterol, saturated fats, cholic acid, and thiouracil sought to consolidate and accelerate the establishment of sustained dyslipidemia and arterial lipid accumulation, promoting the progression towards more advanced atherosclerotic lesions [13]. Besides the implication of atherosclerosis in cardiac ischemia or stroke, disturbance of the structure of the vascular wall may result in increased blood pressure, and other factors such as hyperglycemia and hypertriglyceridemia may contribute to metabolic syndrome [17].

Different atherogenic diets tested in this study had some important effects worth highlighting. Group Athero 1 presented severe clinical manifestations, suggesting that the rats suffered vitamin D2 poisoning, since the signs observed in the rats are consistent with those reported in previous research on the toxicity of vitamin D in Wistar rats at doses of 2 mg/kg. These studies reported that vitamin toxicity caused an uncontrolled increase in plasma calcium and, therefore, produced a mineralization of tissues, organs, cardiac muscle, and blood vessels, leading to structural damage with an eventual decrease in functional capacity [12,18]. In subsequent atherogenic diets 2, 3, and 4, the amounts of the components of the formulations gradually decreased. Although in group Athero 2 the effect was delayed, severe clinical manifestations like those of group 1 were also observed. This suggests that the concentration of vitamin D was still high in group Athero 2; however, in the case of groups Athero 3 and 4 there were no severe clinical manifestations.

On the other hand, speaking of the cardiovascular changes that could be highlighted, a decrease in HR was observed in group Athero 3 on day 60; however, these values are within the range of normal values reported for Wistar rats (300–500 beats per minute, at rest) [19]. In addition, SP and DP decreased over time, with the greatest decrease in week 8. To explain the behavior of blood pressure in the atherogenic model induced in this study, we found reports that arterial stiffness caused by atherosclerotic plaques triggers variations in blood pressure [20]. In humans, a relationship has been observed between the stiffness of the large arteries associated with atherosclerosis and low blood pressure, mainly diastolic pressure [21,22].

Likewise, a relationship between proteins and atherosclerosis has been documented, which is also associated with impaired blood pressure control. Physiologically, the baroreceptors, mainly located in the carotid bifurcation and in the aortic arch, detect blood pressure by responding to the stretching of the arterial wall, sending nerve signals to brainstem centers that regulate the autonomic control of blood pressure. However, when blood vessels are too stiff to stretch, baroreceptor function is impaired. Stiffening of the arteries affects the stretching of the baroreceptors, which leads to decreased nerve input to the autonomic control centers of the brainstem and, therefore, decreased autonomic stimulation of the cardiovascular system [23,24].

After in vivo measurements, the characterization and classification of atherosclerotic lesions was carried out based on morphological observations of the aortic rings. Histological analysis was insufficient to reach a conclusion in groups Athero 1 and 2, due to the loss of experimental animals induced by diet effects. In the case of group Athero 4, the rats developed mild atherosclerotic lesions on day 30 and they remained until the end of treatment on day 60. Therefore, the diet of this group was not suitable for developing a progression model. Finally, in group Athero 3, the rats developed mild lesions on day 30 and severe lesions on day 60, so the atherogenic diet supplied to this group was ideal to develop a model of atherosclerosis progression, achieving the main goal of this study. Once the ideal composition of the atherogenic diet formulation was found to obtain a model of atherosclerosis progression, we performed tests to demonstrate the reproducibility of the model, so a new experiment was carried out again supplying the atherogenic diet 3. The histological results revealed and again confirmed the development of mild lesions on day 30 and severe lesions on day 60. This supports the reproducibility of the model proposed in this study. Additionally, immunohistochemical analysis using CD45 and CD68 markers confirmed the presence of an inflammatory response in the endothelial surface and the tunica adventitia during the phase of atherosclerosis with moderate lesions. In contrast, in the phase of atherosclerosis with severe lesions, the expression of these inflammatory markers decreased and became localized to the areas of atherosclerotic plaque. Tissue labeling with alpha-SMA showed increased expression in the phase with mild lesions, whereas expression decreased in the phase of severe atherosclerosis. These findings align with data reported in human coronary arteries exhibiting varying degrees of atherosclerotic lesions [25]. Another recent study documented a high expression of CD68 by MYH11+ SMCs in the intima layer in the early stage of atherosclerosis in humans [26]. This consistency with human studies supports the validity of the atherosclerosis model developed in our study for investigating the progression of atherosclerosis.

The literature shows that it has been complicated to have a model of atherosclerosis progression as demonstrated in previous studies by Anandhi et al. [7], Gao et al. [9], and other studies [2,5,16,27,28]. The increase in lipid profile markers in our model is clearly associated with the degree of atherosclerotic lesions, in addition to the fact that these characteristics indicate an increased transport of cholesterol and triglycerides from the liver to extrahepatic tissues to be absorbed by these tissues, thus causing atherogenic dyslipidemia. These results coincide with those reported by some authors who have shown that diet-induced hypercholesterolemia results in depressed systolic and diastolic functions due to the increase in the amount of cholesterol in the cardiac sarcolemma, alterations in calcium permeability, and/or the activity of SERCA-218. Therefore, high fat intake predisposes to elevated atherogenic lipoproteins and metabolic syndrome, triggering a proatherogenic lipid profile [29]. In our experiments, HDL levels gradually increase over time; this effect is contrary to the decrease observed in humans in cases of dyslipidemias. This discrepancy is due to differences in lipid metabolism between humans and rats—HDL is the main circulating lipoprotein in rats while in humans that is LDL. This explains the increase in HDL observed in this study by enhancing reverse cholesterol transport mediated by the HDL pathway [29,30,31].

On the other hand, increased lipid concentrations can trigger other metabolic alterations. Thus, in this study the atherosclerotic groups showed a significant increase in glucose levels and partially coincided with the levels of the lipid profile. These results can be explained with data from previous studies, where it is mentioned that increased lipid concentrations are associated with increased plasma glucose levels, since the intracellular accumulation of triglycerides increases endogenous glucose production, which precedes a reduction in glucose tolerance associated with a lower basal uptake of glucose stimulated by insulin [32,33].

Other metabolic alterations that have also been reported in humans are the association between carotid and coronary atherosclerosis with fatty liver, which can be induced mainly by metabolic syndrome, obesity, and diabetes mellitus [34,35,36]. For this reason, in this study serum hepatic markers were measured, which showed an increase with treatment time that may be related to the atherosclerotic degree of the lesions detected in each group. This increase suggests hepatocellular alterations that coincide with what was reported in rats, where the administration of a high-fat diet increased the levels of liver enzymes, relating it to non-alcoholic fatty liver disease [28,37]. There is an important link between fatty liver and atherosclerosis, and it is complicated to evaluate the cause–effect relationship between these diseases; the liver has been reported to be both a target and a determining factor in systemic inflammatory changes. Thus, in atherogenesis, the liver is a target of the resulting systemic abnormalities and, in turn, a source of proatherogenic molecules that potentiate arterial damage [37].

In patients with impaired hepatic metabolism compared to normal subjects, vasodilatation mediated by altered blood flow, increased medial thickness of the intima of the carotid artery, and a higher prevalence of carotid atherosclerotic plaques have been found [37]. This information suggests another mechanism that could explain the decrease in blood pressure observed in this study.

The Paigen diet, while effective for inducing atherosclerosis in rats, has drawbacks associated with the simultaneous induction of metabolic comorbidities, such as hepatic alterations, dyslipidemia, hyperglycemia, and potential hemodynamic changes, which may complicate the isolated interpretation of atherogenic mechanisms. Furthermore, this study is limited to the use of Wistar rats, meaning the findings cannot be directly extrapolated to other strains or species without additional studies to validate their applicability. Additionally, concerns regarding calcium deposition in the plaques must be considered, as the observed gaps during the decalcification of histological sections could be due not only to the presence of calcium but also to artifacts caused by mechanical deformation of the tissue.

## 5. Conclusions

This study provides a new model developed in Wistar rats to study the progression of atherosclerosis induced by an atherogenic Paigen-type diet. To achieve this goal, a new atherogenic formulation was standardized, which is low cost, easy to prepare, and suitable for rats. Our model generates cardiovascular and metabolic changes which are comparable to mild and severe lesions of atherosclerosis. This model is very important for future research to be designed and carried out to better understand and elucidate all the mechanisms involved in each stage of the development of atherosclerosis.

## Figures and Tables

**Table 1:** Formulation of atherogenic diet: phases and composition of the diet for each of the study subgroups.

Time	Phases	Components	Healthy Control	Athero 1	Athero 2	Athero 3	Athero 4
12 days	hypervitaminic	vitamin D2	N/A	240,000 IU	160,000 IU	106,667 IU	80,000 IU
		cholesterol	N/A	30 mg	20 mg	13.33 mg	10 mg
		olive oil	N/A	1.5 mL	1.5 mL	1.5 mL	1.5 mL
48 days	hyperlipidic	standard food	ad libitum	500 g (58.5%)	500 g (67.9%)	500 g (76%)	500 g (80.9%)
		cholesterol	N/A	37.5 g (4.4%)	25 g (3.4%)	16.67 g (2.53%)	12.5 g (2%)
		thiouracil	N/A	2.25 g (0.25%)	1.5 g (0.2%)	1 g (0.15%)	0.75 g (0.1%)
		cholic acid	N/A	15 g (1.75%)	10 g (1.35%)	6.67 g (1.02%)	5 g (0.8%)
		butter	N/A	300 g (35.1%)	200 g (27.15%)	133.33 g (20.3%)	100 g (16.2%)

Note: N/A: not applicable. IU: international units.

**Table 2:** Clinical manifestations observed during the protocol in each group.

	Healthy Control *n* = 16	Athero 1 *n* = 16	Athero 2 *n* = 16	Athero 3 *n* = 16	Athero 4 *n* = 16
weight	increased	decreased	decreased	increased	increased
hair	white	yellowish and greasy	yellowish and greasy	light yellowish	white
physical activity	normal	lethargy	decreased	normal	normal
food and water intake	normal	decreased until it reached zero in the second week	decreased to very low levels for some rats	decreased	normal
diarrhea in rats	N/A	eight	three	N/A	N/A
epistaxis in rats	N/A	eight	five	one	N/A
euthanized rats	none	eight	five	one	none

Note: N/A: not applicable.

**Table 3:** Arterial wall thickness.

	Healthy Control *n* = 8	Atherogenic Diet/Mild Lesions *n* = 15	Atherogenic Diet/Severe Lesions *n* = 19
wall thickness	95.74 μm	171.8 μm	175.1 μm
standard error	1.67	14	11.8
*p* value		0.0023 *	0.0009 * 0.9780 ^†^

* Statistically significant regarding the healthy control. ^†^ Statistical analysis showed no difference between mild lesions and severe lesions.

**Table 4:** Biochemical parameters in healthy and atherosclerotic rats.

	Healthy Control *n* = 8	Atherogenic Diet/Mild Lesions *n* = 8	Atherogenic Diet/Severe Lesions *n* = 8
total cholesterol (mg/dL)	55.56 ± 1.28	113.8 ± 4.69 *	127.5 ± 8.16 *
LDL-C (mg/dL)	38.78 ± 2.5	103 ± 2.75 *	123 ± 12.97 *
HDL-C (mg/dL)	22.16 ± 1.27	27.41 ± 1.38	33.66 ± 2.6 *
triglycerides (mg/dL)	30.38 ± 1.88	40.41 ± 1.23 *	73.7 ± 10.23 * ^†^
glucose (mg/dL)	98.3 ± 4.96	159.4 ± 16.7 *	134.2 ± 3.96 *
AST (U/L)	114.9 ± 8.25	173.9 ± 12.46 *	253.9 ± 37.25 * ^†^
ALT (U/L)	58.11 ± 7.59	117 ± 14.3	272.6 ± 58.73 * ^†^
LDH (U/L)	1122 ± 62.16	1259 ± 151.5	1499 ± 90.61 *
ALP (U/L)	47.08 ± 4.35	110.4 ± 11.67 *	120.3 ± 15.71 *

Note: * Statistically significant regarding the healthy control (*p* < 0.05). ^†^ Statistically significant regarding the mild lesions (*p* < 0.05). The values are expressed as the means ± SEM (*n* = 8 for each group). Intergroup analysis: *t* test. Abbreviations: Low-density lipoprotein cholesterol (LDL-C), High-density lipoprotein cholesterol (HDL-C), Aspartate Aminotransferase (AST), Alanine Aminotransferase (ALT), Lactate Dehydrogenase (LDH), Alkaline phosphatase (ALP).

## Data Availability

The raw data supporting the conclusions of this article will be made available by the authors on request.

## Abbreviations

The following abbreviations are used in this manuscript:

Apo E	Apolipoprotein E
LDLR	Low density lipoprotein receptor
HDL	High-density lipoprotein
LDL	Low-density lipoprotein
LDH	Lactate dehydrogenase
ALP	Alkaline phosphatase
ALT	Alanine aminotransferase
AST	Aspartate aminotransferase
TC	Total cholesterol
SP	Systolic pressure
DP	Diastolic pressure
HR	Heart rate
